# The clinical efficacy of traditional Chinese medicine in the treatment of malignant pleural effusion: A protocol of systematic review and meta-analysis

**DOI:** 10.1097/MD.0000000000022403

**Published:** 2020-09-25

**Authors:** Zhen Lin, Mengyuan Jiang, Lirong Gao, Huachun Zhang

**Affiliations:** Department of Oncology, LongHua Hospital, Shanghai University of Traditional Chinese Medicine, Shanghai, China.

**Keywords:** malignant pleural effusion, protocol, systematic review, traditional Chinese medicine

## Abstract

**Background:**

The objective of this meta-analysis was to summarize and identify the available evidence from studies to estimate the clinical value of traditional Chinese medicine (TCM) in the treatment of malignant pleural effusion (MPE). And provides clinicians with evidence on which to base their clinical decision making.

**Methods:**

This review will include all studies comparing clinical efficacy of TCM in the treatment of MPE. The search strategy will be performed in 9 databases. We will not establish any limitations to language and publication status, published from inception to the July, 2020. Two reviewers will screen, select studies, extract data, and assess quality independently. Outcome is clinical efficacy, QLQ-C30 questionnaire and safety. The methodological quality including the risk of bias of the included studies will be evaluated. We will carry out statistical analysis using RevMan 5.3 software.

**Results:**

This study will summarize current evidence to assess the efficacy and safety of TCM in the treatment of MPE.

**Conclusion:**

The findings of this study will provide helpful evidence for the clinician, and will promote further studies, as well as studying the value of TCM.

## 1 Introduction

Malignant pleural effusion (MPE) is 1 of the commonest causes of an exudative pleural effusion, and its incidence is increasing with increasing cancer prevalence and as more effective cancer therapy that prolongs life. It is the commonest cause of a unilateral massive pleural effusion, although 10% to 13% can be bilateral. Median survival after a diagnosis of MPE depends on the underlying malignancy and stage at diagnosis, and varies between 3 and 12 months.^[1–3]^ Most MPEs are secondary to metastases to the pleura from other sites, most commonly lung and breast, which together cause 50% to 65% of all MPE. Breathlessness, dyspnea and other symptoms often seriously distress and affect the quality of life (QOL).^[4–7]^

In recent years, traditional Chinese medicine (TCM) has played an increasingly important role with its unique advantages. Several studies have evaluated its clinical outcomes of TCM in the treatment of MPE. To the best of our knowledge, there is no meta-analysis analysis the clinical efficacy of TCM for MEP. Consequently, the objective of this meta-analysis was to summarize and identify the available evidence from these studies to estimate the clinical value of TCM. And provides clinicians with evidence on which to base their clinical decision making.

## 2 Methods

### 2.1 Study registry

The protocol was registered on the International Platform of Registered Systematic Review and Meta-analysis Protocols (INPLASY202080105). The preferred reporting items for systematic review and meta-analysis protocols (PRISMA) will serve as guidelines for reporting present review protocol and subsequent formal paper.^[8]^

### 2.2 Eligibility criteria for including studies

#### 2.2.1 Types of studies

We will include all researches studying the clinical efficacy of TCM in the treatment of MPE, including observational study and RCT. Any other types of studies, such as animal studies, case reports, case series and review will all be excluded.

#### 2.2.2 Types of interventions

##### 2.2.2.1 Experimental group

All patients in the experimental group received TCM for their treatment in this study (including oral Chinese medicine or external application of Chinese medicine). 2.2.2.2. Control group.

The participants in the control group could receive any other treatments in this study

#### 2.2.3 Types of patients

To be involved in this study, all the patients were required to meet the inclusion criteria as follows:

(1) aged 18 to 80 years;
(2) definite pleural effusion with medium or above amount confirmed by X-ray or ultrasound;
(3) advanced malignant tumor with MPE confirmed by histopathology or cytology; (4) the estimated survival time is more than 3 months;
(4) Karnofsky score ≥ 60, ECOG PS score ≤ 2.

#### 2.2.4 Types of outcome measurements

##### 2.2.4.1 Primary outcomes

The criterion of efficacy was divided into 4 categories refer to WHO standard. Complete Remission (CR): effusion disappears and symptoms are relieved for at least 4 weeks; Partial Remission (PR): the effusion was reduced by more than 50% compared with that before treatment, and the symptoms were relieved and maintained for at least 4 weeks; Stable (SD): the effusion decreased by less than 50% compared with that before treatment, with no increasing trend, and the symptoms partially relieved; Invalid (PD): effusion grows rapidly. The total effective rate was (CR+PR)/ (CR+PR+SD+PD) × 100%.

##### 2.2.4.2 Secondary outcomes

QLQ-C30 questionnaire: QLQ-C30 was developed the European Organization for Research and Treatment of Cancer (EORTC) in 1986. It is widely used to evaluate the quality of life for patients with oncology. The QLQ-C30 incorporates nine multi-item scales: 5 functional scales (physical, role, cognitive, emotional, and social); 3 symptom scales (fatigue, pain, nausea and vomiting); and a global health and quality-of-life scale. Several single-item symptom measures are also included.^[9,10]^

##### 2.2.4.3 Literature sources and search

We will perform literature searches using the following electronic bibliographic databases from their inception onwards to the July, 2020: MEDLINE, Springer, Web of Science, PubMed, EMBASE, the Cochrane Central Register of Controlled Trials, Evidence Based Medicine Reviews, VIP, and CNKI. We will not establish any limitations to language and publication status. The following electronic databases were searched from their inception dates through August 2020. The search terms were integrated as follows: “∗ malignant pleural effusion ∗ AND (∗Traditional Chinese Medicine∗ OR ∗Traditional Chinese Medicine Formula∗ OR ∗Chinese Herb Formula∗ OR ∗Chinese herbal drug∗)”.

##### 2.2.4.4 Study selection

**Figure 1:**
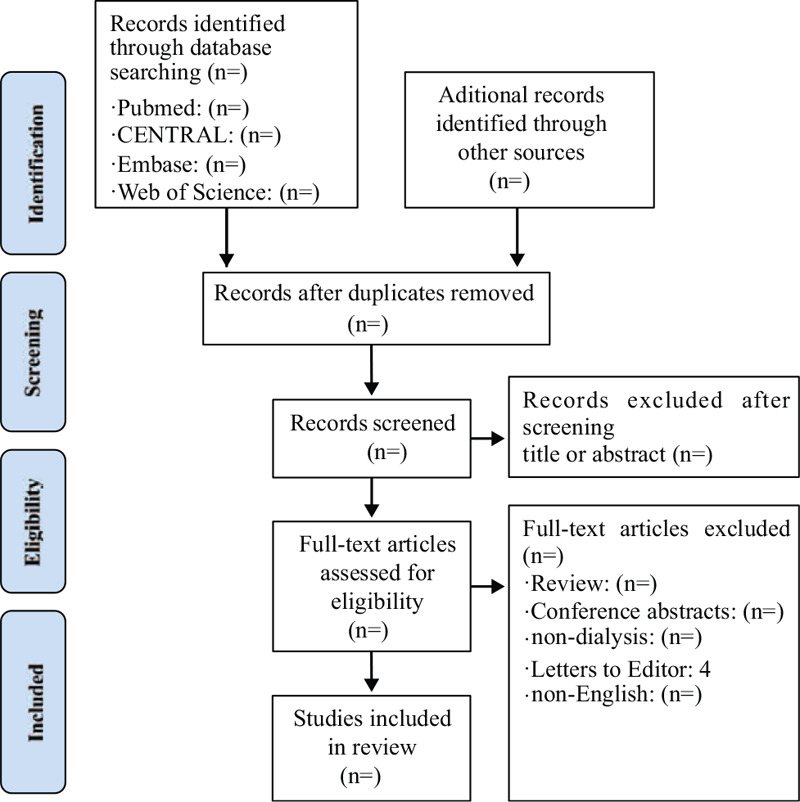
Study flow.

All duplicated studies will be imported into Endnote X7 software and excluded before the screening. Two authors will independently scan all the records from title and abstract and all irrelevant literatures will be removed. Then, full manuscripts of all remaining studies will be further identified to check if they meet all inclusion criteria. We will note all excluded citations with specific reasons. If there are any different opinions between 2 authors, we will invite another author for consultation and final decision will be made after discussion. The detail of the study selection will be presented in a PRISMA flow diagram (Fig. 1)

##### 2.2.4.5 Data extraction

Two authors will independently extract the following associated information from each included trial: first author, time of publication, location, sample size, randomization methods, blinding, concealment, pathologic types, details of intervention and controls, duration of follow-up, outcome measurement tools, and any other relevant information. A third senior author will help to reconcile any divergences between 2 authors.

#### 2.2.5 Missing data dealing with

If we identify any unclear or missing data, we will contact original authors to obtain them. If we cannot get reply, we will only analyze available data and will discuss its potential affect as limitation.

#### 2.2.6 Quality assessment

Two independent reviewers assessed the methodological quality by using the Newcastle-Ottawa Scale with some modifications to match the needs of this study.^[11,12]^ The quality was evaluated by examining 3 items: selection, comparability, and exposure, with higher scores representing studies of higher quality. The quality of each study was graded as either level 1 (0–5) or level 2 (6–9).^[13]^ This review also assessed the clinical heterogeneity to evaluate whether the trials were similar enough to pool data.

#### 2.2.7 Subgroup analysis

We will preside over subgroup analysis to explore any potential heterogeneity and inconsistency based on the treatment

#### 2.2.8 Sensitivity analysis

We will consider running sensitivity analysis to identify the robustness and stability of merged results by excluding studies with high risk of bias.

#### 2.2.9 Reporting bias

If necessary, we will examine the reporting bias using funnel plot and Egger regression test when >10 trials are included.

### 2.3 Data synthesis

We will undertake RevMan 5.3 software to analyze data and to perform meta-analysis if it is necessary. We will calculate all continuous data using mean difference or standardized mean difference and 95% confidence intervals. As for dichotomous data, we will exert it using risk ratio and 95% CI. The heterogeneity as determined by the Cochran statistics was <0.10 of the χ^2^ test. If the *I*^2^ value was >50%, we marked it as a considerable level of heterogeneity; otherwise, we considered it to be a good homogeneity. We also assessed clinical heterogeneity. Statistically and clinically homogeneous studies were pooled using a fixed-effects model; otherwise, a random-effects model was used when the heterogeneity was significant. Additionally, subgroup analysis will be operated to explore any possible reasons for the high heterogeneity. Whenever it is possible, we will conduct meta-analysis if at least 3 eligible criteria are fulfilled. Otherwise, meta-analysis will not be carried out if only 1 or 2 studies meet the inclusion criteria. Under such situation, the findings will be presented in a narrative summary. We will perform narrative synthesis if running meta-analysis is inappropriate due to the high heterogeneity. All narrative descriptions will be carried out based on the Guidance on the Conduct of Narrative Synthesis in Systematic Reviews.

## 3 Discussion

MPE is mainly caused by lung cancer or other chest malignant tumors. About 50% of lung cancer or breast cancer patients will have pleural effusion in the course of disease. Once MPE appears, it indicates that the primary tumor has local focus or adjacent important organ metastasis, and the chance of radical operation is lost. According to statistics, the median survival time of patients with MPE is only 3–12 months. MPE often grows rapidly and aggravates asthma which patients can seldom tolerate. In severe cases, it may be life-threatening. Therefore, for patients with MPE, it is particularly important to prolong their survival period and improve their quality of life.

The treatment of MPE with combination of TCM and western medicine has been widely recognized. It is not only superior to simple use of western medicine, but also has advantages in control of adverse reactions. TCM has certain characteristics and advantages in the treatment of MPE. It can enhance immunity, relieve symptoms, improve life quality, and has little side effects, which is easy for patients to accept. It can be the first choice especially for those who cannot receive chemotherapy. However, there is few relevant clinical report of large cases.^[14–16]^

The strength of this systematic review and meta-analysis will include: search a comprehensive range of databases, including Chinese and English databases, more rigorous and detailed concerning quality assessment and data extraction. In addition, the findings obtained in the present study will provide helpful evidence in clinical practice. Furthermore, it will also help to promote further studies and clarify the direction for the future research.

On the contrary, this study has several potential limitation. There may be a language bias, although there is not language limitation in this study. Moreover, there may be a large heterogeneity, which may bias the results.

## Author contributions

LZ, MYJ, and LRG conducted of the protocol and drafting the manuscript. All authors participated in the design of the study. HCZ is co-corresponding author of this manuscript. All authors read and approved the final manuscript.

**Conceptualization:** Huachun Zhang.

**Data curation:** Zhen Lin, Mengyuan Jiang, Huachun Zhang.

**Funding acquisition:** Huachun Zhang.

**Methodology:** Lirong Gao.

**Software:** Zhen Lin.

**Supervision:** Zhen Lin.
